# Emerging Role of Microbiome in the Prevention of Urinary Tract Infections in Children

**DOI:** 10.3390/ijms23020870

**Published:** 2022-01-14

**Authors:** Anna Kawalec, Danuta Zwolińska

**Affiliations:** 1 Clinic of Pediatric Nephrology, University Hospital, 50-556 Wroclaw, Poland; 2 Department of Pediatric Nephrology, Wroclaw Medical University, 50-556 Wroclaw, Poland; danuta.zwolinska@umed.wroc.pl

**Keywords:** microbiome, urinary tract infection, pediatric diseases

## Abstract

The microbiome of the urinary tract plays a significant role in maintaining health through the impact on bladder homeostasis. Urobiome is of great importance in maintaining the urothelial integrity and preventing urinary tract infection (UTI), as well as promoting local immune function. Dysbiosis in this area has been linked to an increased risk of UTIs, nephrolithiasis, and dysfunction of the lower urinary tract. However, the number of studies in the pediatric population is limited, thus the characteristic of the urobiome in children, its role in a child’s health, and pediatric urologic diseases are not completely understood. This review aims to characterize the healthy urobiome in children, the role of dysbiosis in urinary tract infection, and to summarize the strategies to modification and reshape disease-prone microbiomes in pediatric patients with recurrent urinary tract infections.

## 1. Introduction

In recent years, much attention has been paid to the human microbiome and its importance in health and disease [1,2,3,4]. It is estimated that the human body is colonized by 10–100 trillion microbial cells containing 100-fold more genes than the human genome [5]. The term ‘microbiota’ refers to the group of microorganisms associated with a specific biologic niche, mainly bacteria, but also protozoa, viruses, fungi, and archaea [2,6]. Correspondingly, the term ‘microbiome’ relates to the group of microbial genomes in a specific environment [2,6]. 

Urobiota helps maintain bladder homeostasis in terms of maintaining the integrity of the urinary tract epithelium, protecting against infections, regulating neurotransmission, and promoting the proper functioning of the immune system [7]. 

The composition, characteristics, and role of the microbiota of the urinary tract are still under investigation [7,8]. The majority of the urobiome studies have focused on the adult population, and the alterations of the microbial community structure have been linked to several urological diseases such as urinary tract infections, incontinence, overactive bladder, urolithiasis, and prostate or bladder cancer [9,10]. The number of studies in the pediatric population is rather limited and involved smaller numbers of subjects, thus the characteristic of the urobiome in children, its role in a child’s health, and pediatric urologic diseases are not completely understood [11]. 

The aim of this article is to present the urobiome of healthy children depending on the age, the role of dysbiosis with a focus on urinary tract infections. Another goal is to summarize the results of recent studies regarding the modification of the urobiome in the prevention of recurrent urinary tract infections in children.

## 2. The Urobiome Investigation

### 2.1. Diagnostic Techniques

From the 1950s, the urinary tract was believed sterile under normal conditions. This approach has changed with the start of the Human Microbiome Project, the first large-scale mapping of the human microbiome using culture-independent methods. Standard urine cultures detect aerobic and fast-growing bacteria, while slow-growing anaerobic microorganisms or bacteria that require different growth conditions remain undetected [12,13]. 

Relatively recently, due to advances in bacterial assessment, it has been shown that the bladder is not a sterile environment [12]. In particular, new diagnostic techniques such as PCR, expanded quantitative urine culture (EQUC), whole-genome sequencing (WGS), and next-generation sequencing (NGS) including 16S ribosomal RNA gene sequencing allowed the discovery of uncultured microorganisms and revealed the existence of a urinary microbiome [12,13,14]. 

### 2.2. Challenges in the Urobiome Research

Although new diagnostic approaches have facilitated a qualitative and quantitative identification of individual bacteria species with very high accuracy, there are some significant concerns due to the limitations of the methodology, the low biomass of the urinary microbiota, and its immediacy to other bacterial niches with higher microbial biomass [12,14]. The techniques that have been used to obtain urine samples to investigate the urinary microbiota include the midstream clean-catch urine, transurethral catheterization, or suprapubic aspiration [14,15]. The choice of a specific method of a urine sample collection may affect the obtained results [14] with difficulties in distinguishing bacteria from the bladder from microbial contamination from the skin, vulvovaginal and perineal flora [12,14,15]. Additionally, urethral or bladder tissue samples, obtained by urethral swabs, bladder biopsy, or scraping, might be used in urobiome research [16]. Pohl et al. showed that the urobiome of healthy men and women differs according to sampling method (voiding versus catheterized samples). In addition, their results indicate that the microbiome of the urethra and bladder are different. In the urethral samples, the most abundant genera were *Veillonella*, *Staphylococcus*, and *Neisseria*, while in bladder samples *Lactobacillus*, *Streptococcus*, and *Gardnerella* [17]. According to Wolfe et al., suprapubic aspiration sampling minimizes contamination from non-urinary sites and provides the best view of the bladder bacteria [18]. The recommended sampling method to use in studies investigating the microbiome of the urinary tract is transurethral catheterization [18,19]. However, the use of invasive techniques to collect urine for urobiome research in the pediatric population raises an ethical concern. It is worth underlining that urine cultures of clean-catch urine samples have good efficacy in diagnosing urinary tract infections in children [20,21], and the contamination rate of urine collected via urethral catheterization is lower but not significantly different from that of clean-catch [21]. Interestingly, Bundgaard-Nielsen et al., who investigated the impact of the collection method on the urobiome composition, did not report any interpersonal daily or day-to-day deviations in microbiota composition in women, girls, or boys [22].

Another methodological challenge is the inability of the sequencing technique to differentiate DNA from living versus dead bacteria [11,12]. To assess the viability of identified microorganisms, modified clinical cultivation procedures were implemented, and an enhanced quantitative urine culture (EQUC) protocol, which uses multiple culture media and various incubation conditions to cultivate bacteria that do not grow under standard conditions, was developed [15,18,19]. Considering a non-invasive urine collection method for the urobiome studies, Ozer et al. using first voided or midstream urine samples showed no significant difference regarding 16s ribosomal RNA analysis [23].

Furthermore, sequencing of 16S ribosomal RNA gene cannot provide information below the species level, in particular data about the strain level or presence of virulence factors. These details should be taken into consideration, as not all strains belonging to the same species exhibit pathogenic behavior [24]. For instance, several virulence genes are characteristic of uropathogenic *Escherichia coli* (*E. coli*) and are not present in other *E. coli* strains. These genes encode virulence factors essential in UTI development, such as adhesins (*fimH* gene encoding type 1 fimbrial tip adhesin), siderophores, and toxins (*hlyA* gene encoding α-hemolysin, *cnf1* gene encoding cytotoxic necrotizing factor) [25,26].

## 3. The Urobiome Origin and Composition

### 3.1. The Origin of Urinary Microbiota

The origin of urinary microbiota is still not entirely clear. A urinary microbiome was identified even in neonates [27]. The fetus is considered sterile during normal pregnancy and acquires bacteria through transmission from the mother at delivery [28]. The maternal microbiome has a strong influence on the neonatal skin, oral mucosa, and nasopharyngeal microbiome development, and might similarly influence newborns’ urobiome [29]. The composition and function of the early infant microbiota are primarily determined by birth mode, maternal microbiota, exposure to antibiotics, and feeding practices in early life [30]. It is hypothesized that the main origins of urinary microbiota are microbial communities of the gastrointestinal tract and vagina, majorly because of the anatomical proximity. Evidence suggests that the infant microbiota assembles and stabilizes 2–3 years after birth [28,30]. In adolescents, pubertal hormonal changes may influence the maturation of the microbiome of the urinary tract [11]. However, the effect of age on the urobiome of healthy children has been scarcely analyzed. 

### 3.2. Urobiome Composition Depending on the Child’s Age 

Kinneman et al. aimed to examine the urinary microbiome of eighty-five children younger than 48 months who presented to the Emergency Department mainly because of fever. Urine samples were collected via transurethral catheterization. The urinary microbiome was identified in every child. The most abundant families detected in urine samples were tissierellaceae, prevotellaeae, veillonellaceae, enterobacteriaceae, and comamonadaceae, while the five most abundant genera were *Prevotella*, *Peptoniphilus*, *Escherichia*, *Veillonella*, and *Finegoldia*. There were no significant differences according to gender, probably because urine samples were collected via catheterization, which minimized periurethral and perineal contamination. Nine children were diagnosed with urinary tract infection, in this group decreased urobiome diversity was observed [27]. 

Two studies investigated the urobiome in toilet trained children [31,32]. Fredsgaard et al. described the voided urinary microbiota in a group of thirty asymptomatic prepubertal children aged 6 to 10 years old. Bacterial DNA was extracted from all “clean-catch” midstream urine samples. The voided urinary microbiota differed significantly according to gender. The urine of girls was dominated by *Prevotella* (18.2%), *Porphyromonas* (12.9%), *Ezakiella* (8.1%), *Prevotella* (7.4%), and *Dialister* (7.0%). In boys, the most abundant genus was *Porphyromonas* (22.4%), followed by *Ezakiella* (12.0%), *Campylobacter* (11.6%), *Prevotella* (8.6%), and *Dialister* (3.7%). The authors suggest that the discrepancy in the urobiome composition between girls and boys could be due to gender-specific anatomy and differences in sex hormones levels in the prepubertal period [31].

Another study conducted by Kassiri et al. focused on prepubertal boys aged from 3 months to 8 years. The catheterized urine samples did not reveal a clear predominance of a particular bacterial genus. The majority of urine samples showed the presence of *Staphylococcus* and *Varibaculum* species, and to a lesser extent *Peptoniphilus* and *Actinobaculum*. The authors support the opinion that the development of the urinary microbiota starts and evolves in early life and becomes more stable in adulthood, similarly to the microbiota of the gastrointestinal tract. However, the study group consisted of patients who required elective urologic procedures, therefore the composition of urobiome may not adequately represent the bacteria present in the urinary tract of children without urological pathologies [32].

From puberty, the shift in the composition of the microbiota of the urinary tract may reflect physiological changes. Presumably, the urobiome of adolescents begins to resemble the urobiome of healthy adults. However, no studies were performed on this topic, and further investigations regarding alterations of the urinary microbiota in this age group are needed.

## 4. Dysbiosis and Urinary Tract Infection

An imbalance in urinary microbiota may cause an overgrowth of pathogenic bacteria. Changes in urobiome composition may impact disease susceptibility and pathophysiology [3]. Dysbiosis of the urobiome has been related to an increased risk of urinary tract infection, nephrolithiasis, and dysfunction of the lower urinary tract [9,10,33]. Although most studies focused on adults, the number of investigations evaluating the relationship between the urobiome, dysbiosis, and urinary tract diseases in children increases [11,24,34,35]. 

The findings of recent studies in this field may have significant clinical implications. Strategies of manipulating and reshaping disease-prone microbiomes may serve as an alternative or supportive option in the management of pediatric urological diseases. The modifications of the urobiome include microbial supplements (probiotics or synbiotics), foods or substrates (diet or prebiotics), microbial suppression or elimination (antibiotics) strategies [3].

Urinary tract infection is one of the most common bacterial infections in children [36]. The predominant pathogen is *E. coli* which accounts for 90% of primary UTIs in girls and 80% of primary UTIs in boys [36,37,38,39]. Other bacterial pathogens include gram-negative *Klebsiella*, *Enterobacter*, *Proteus* and *Citrobacter* [37,38], and gram-positive *Enterococcus* and *Staphylococcus saprophyticus* [38]. The clinical manifestation of UTI has a broad spectrum and may present as infection of the lower or upper urinary tract. According to patient comorbidities, UTIs can be divided into uncomplicated and complicated. In children, complicated UTIs are mainly associated with congenital anomalies of kidneys and urinary tract. A serious problem is the recurrence of urinary tract infections, defined as three or more infections per year [40]. Noteworthy, different microbial communities are associated with UTIs in specific patient groups (Table 1). 

Most UTIs result from ascending infection, and periurethral colonization with uropathogenic bacteria is the first step in the development of UTI [38]. Among several host defense mechanisms, a diverse urobiome has been associated with a protective role. According to Kinneman et al., children with UTIs had a significantly decreased alpha diversity, and the composition of the microbiome clustered separately compared with children without UTIs [27]. There is no consensus regarding the taxonomy of healthy urobiota. Additionally, the proportion of species forming the urobiome is dynamic. Commensal bacteria of the urinary tract are believed to serve as a barrier for uropathogens by blocking access to urothelium, producing antimicrobial compounds, or out-competing pathogenic bacteria for common resources [42]. It is suggested that probably most bacteria causing UTIs are part of the resident urinary tract bacteria and reveal their uropathogenicity due to an imbalance in normal urobiome composition [43]. UTIs develop as a result of several host-microbial interactions, and host susceptibility and bacterial virulence factors are crucial elements of that interplay. The interaction between the uroplakin receptors expressed at superficial urothelial cells and bacterial type 1 fimbrial adhesin fimH may serve as an example [43]. The process of *E. coli* invasion and formation of an intercellular population of bacteria starts after uropathogenic *E. coli* fimbriae connect with uroplakin Ia/Ib [44]. 

The reservoirs of uropathogens are the gastrointestinal tract and vagina [45,46]. Paalanne et al. conducted a case-control study that assessed differences in the gut microbiome between children with UTI and healthy controls. The biodiversity of the intestinal microbiome of children was similar in both groups, but there were differences at the family and genus levels. The genus *Enterobacter* was more abundant in the UTI patients, and the family peptostreptococcaceae was more abundant in controls. These findings suggest that the intestinal environment and its microbial community are associated with the risk of UTI in the pediatric population [47]. 

Similarly, the vaginal microbiota might impact host susceptibility to UTI and can either protect against or increase the risk of UTIs in girls. Gorbachinsky et al. demonstrated that vulvovaginitis may cause UTIs by changing the perineal microbiome and increased colonization of uropathogens [48]. Previous studies indicated that the vaginal microbiome alters with age. In prepubertal age, a variety of anaerobes, diphtheroids, coagulase-negative staphylococci, and *E. coli* dominate, while the postmenarcheal vaginal microbiome is dominated by *Lactobacillus* spp. [11,29]. In contrast, a prospective longitudinal study among perimenarcheal girls conducted by Hickey et al. demonstrated that bacteria producing lactic acid were dominant in the vaginal microbiota of most girls well before the onset of menarche [49]. A protective effect of *Lactobacillus* spp. is associated with the following mechanisms: maintaining the vagina’s characteristic low pH (mainly due to lactic acid production), releasing antimicrobial compounds such as hydrogen peroxide and bacteriocins, out-competing pathogenic bacteria, preventing the adhesion of pathogens to epithelial cells, and modulation of the host immune system by activation of the Toll-like receptor pathway and interleukins production [11,29,50,51].

## 5. Modification of the Microbiome in the Prevention of Urinary Tract Infection in Children

It has been shown conclusively that there is a connection between dysbiosis in the microbiome and UTIs [39]. Several interventions have been studied for preventing UTIs, including antibiotic treatment and non-antibiotic measures. Antibiotic prophylaxis attempts to prevent UTIs in children who are at a higher risk, such as those with vesicoureteral reflux and hydroureteronephrosis [52]. The literature demonstrated that long-term antibiotic prophylaxis did not reduce the frequency of UTI or prevent renal scarring, but increased the risk of antibiotic resistance development, impacted the microbiome, and had potential long-term side effects [35,52]. According to the updated Cochrane Systematic Review by Williams and Craig, antibiotics may make slight or no difference to the risk of recurrent urinary tract infection compared to placebo or no treatment (RR = 0.75, 95% CI: 0.28–1.98). Furthermore, the risk of a UTI caused by resistant bacteria was almost 2.5 times higher in patients on antibiotic prophylaxis than for those on placebo or no treatment (RR = 2.40, 95% CI: 0.62–9.26) [53].

Because of the growing rate of resistant bacteria and the increase in the incidence of UTIs caused by multi-drug resistant microorganisms, effective non-antibiotic methods are searched. New treatment approaches aiming to reshape the disease-prone microbiomes include dietary supplementation, probiotics, intravesical instillations of probiotics or glycosaminoglycans, inoculation with less-pathogenic bacteria, immunostimulants, and vaccines [51].

### 5.1. The Impact of the Child’s Diet on the Urobiome

#### 5.1.1. Breastfeeding

Breastfeeding protects children from several infectious diseases, and epidemiological evidence indicates the reduced UTI incidence in breast-fed infants. The protective effect of breast milk is assigned to its impact on the gastrointestinal flora and its essential role in normal infant microbiota assembly [30]. Additionally, high levels of immunoglobulin A in breast milk prevent bacterial adherence to the intestinal mucosa and urothelium [54]. Another possible protective effect may result from the presence of the human milk oligosaccharides (HMOs) that are indigestible and in unchanged form reach the colon, where they function as prebiotics by promoting the growth of beneficial bacteria such as *Bifidobacteria* and *Lactobacilli* and help to establish a healthy gut microbiota [55,56]. In addition, HMOs serve as soluble decoy receptors for surface adhesins of various bacteria, and by competitive binding to pathogens they inhibit bacterial adhesion to urothelium [30,55,57]. Lin et al. found that HMOs can protect bladder epithelial cells from cytotoxicity and proinflammatory effects caused by uropathogenic *E. coli* (UPEC) [57]. However, Ardiç and Yavuz conducted a prospective cohort study and did not show any correlation between exclusive breastfeeding and duration of a breastfeeding period and the number of UTI episodes within five years [58].

#### 5.1.2. Cranberry Products

Dietary supplementation in UTI prevention includes usage of cranberry products which can potentially influence the composition of the urobiome. Cranberries contain several agents inhibiting the adhesion of *E. coli* to urothelial cells receptors. These compounds are fructose which prevents the adhesion of *E. coli* with type 1 pili, and proanthocyanidins which affect the adherence of *E. coli* with P pili. Anthocyanidins and proanthocyanidins are tannins (polyphenols) known for their anti-microbial function and are considered the most clinically relevant in UTIs prevention [51]. Cranberry is safe when taken orally, but consumption in large amounts might result in diarrhea. It should be used with caution in patients with nephrolithiasis [59]. According to recently published systematic reviews and meta-analyses, cranberry products reduce the risk of recurrent UTIs in the pediatric population [60,61]. It was shown that cranberry as adjuvant therapy significantly decrease the recurrence rate of UTI in susceptible groups of children (RR=0.55; 95% CI: 0.31–0.97) [60]. Similar conclusions draw Meena et al., who focused on non-antibiotic interventions in UTI prevention in children. The analysis of five randomized controlled trials including 466 participants compared cranberry products with placebo in children with the structurally normal urinary tract and showed that cranberry products significantly reduced the incidence of recurrent UTI (RR = 0.48; 95% CI: 0.28–0.80) over a period of 6–12 months in comparison to placebo or no therapy. Interestingly, one trial conducted among 192 children reported that cranberry was as effective as antibiotic prophylaxis in patients with recurrent UTIs (RR = 0.92; 95% CI: 0.56–1.50) [61].

### 5.2. Probiotics

Modulation of urobiome with probiotics is promising in the prevention of recurrent UTIs in children. Probiotics are “live microorganisms that, when administered in adequate amounts, confer a health benefit on the host” [62]. A beneficial effect in the management of UTIs has been observed for *Lactobacillus rhamnosus*, *Lactobacillus acidophilus*, *Lactobacillus fermentum*, *Lactobacillus reuteri*, *Bifidobacterium bifidum*, and *Bifidobacterium lactis* [9,63,64]. Probiotics can be administered orally, vaginally, or as intravesical instillation. There is no consensus regarding the selection of probiotic strains, accurate dosage, mode of delivery, or length of therapy. The literature reports inconsistent results of the effectiveness of probiotics in UTI prevention in children, which might result from methodology differences. Additionally, factors influencing the viability of probiotic bacteria during production, storage, and delivery until consumption time, including temperature, pH, molecular oxygen, and additives such as sugar, sodium chloride, and antimicrobial preservative, may affect the clinical outcomes. Furthermore, the effects of probiotic interventions are attributable to a specific strain and might be absent in other strains of the same bacteria [51,63,64].

#### 5.2.1. Oral Probiotics

The Cochrane Systematic Review by Schwenger demonstrated no significant difference between the use of probiotics compared with placebo or no treatment in UTI prevention in children. However, the authors underlined that a beneficial role of probiotics cannot be excluded, mainly because the data were few and derived from small studies with poor methodological reporting [65]. Similar conclusions draw the meta-analysis by Hosseini et al., which revealed that probiotics in monotherapy did not reduce the incidence or recurrence of UTI in children, whereas their use as adjuvants to antibiotics decreased the incidence rate of UTIs [66].

In contrast, the recent systematic review and meta-analysis by Meena et al. showed that probiotics not only were more effective than placebo in reducing UTI recurrence in children with no structural anomaly of the urinary tract (RR = 0.52; 95% CI: 0.29–0.94) but also have similar efficacy as antibiotic prophylaxis in preventing UTI recurrence in children with vesicoureteral reflux (RR = 0.82; 95% CI: 0.56–1.21). In children with vesicoureteral reflux, the probiotic therapy significantly lowered the risk of antimicrobial resistance compared to antibiotic prophylaxis (RR = 0.38; 95% CI: 0.21–0.69), without any significant difference in risk of new kidney scarring (RR = 0.60; CI: 0.25–1.44). The authors observed that combined therapy that included antibiotic plus probiotic was more effective than antibiotic therapy alone and, in addition, significantly decreased the incidence of antibiotic resistance [61].

The therapy with oral probiotics is considered to be safe and well-tolerated. The frequency of reported side effects is low. The most commonly reported adverse effects of probiotics therapy include: diarrhea, nausea, vomiting, constipation, and vaginal symptoms [65].

#### 5.2.2. Intravesical or Vaginal Probiotics

Both the gastrointestinal tract and vagina are reservoirs of uropathogens. Probiotics administered orally have been shown to transit through the gastrointestinal tract, resulting in vaginal colonization and affecting the urobiome composition. The number of probiotic microorganisms that can reach the vagina and influence the urobiome after oral administration is lower than direct vaginal or intravesical instillation and depends on the viability of the strains as they pass through the gastrointestinal tract [51]. In the pediatric population, probiotics are mainly administered orally. Probably, instillation of probiotics directly into the bladder or vagina may be more effective in dysbiosis correction and UTIs prevention than oral administration, but it is difficult in small children.

There is a lack of studies reporting the use of vaginal probiotics in girls, however, the preliminary observations in the adults are promising. Stapleton et al. found that intravaginal suppository probiotic containing *Lactobacillus crispatus* (Lactin-V; Osel) effectively prevented recurrence of UTI in premenopausal women. The adverse effects of therapy with intravaginal suppository probiotic included vaginal discharge, itching, or moderate abdominal discomfort [67]. Further studies are needed to confirm the efficacy of intravaginal probiotics in recurrent UTIs prevention. Presumably, this type of intervention could be considered among girls suffering from recurrent UTIs.

Initial results of intravesical instillation of probiotics appear to be encouraging. Groah et al. demonstrated that one or two doses of intravesical *Lactobacillus rhamnosus* GG (LGG) instillation as a reaction to inflammatory urinary symptoms is safe and well-tolerated in adult and pediatric patients with neurogenic lower urinary tract dysfunction due to spinal cord injury or disease who use intermittent catheterization [68]. The results of the studies carried out by Forster et al.’s suggest that intravesical instillation of LGG is a safe route of administration. Reported side effects after *Lactobacillus* intravesical instillation included transient cloudy and malodorous urine in two out of five pediatric participants. The symptoms self-resolved during seven days following intervention [69].

Tractenberg et al. assessed the effects of intravesical LGG on urinary symptoms in patients with neurogenic lower urinary tract dysfunction in an uncontrolled pilot study. The study group consisted of ninety-six adults and seven children with spinal cord injury or disease. Self-instilled LGG appeared to have a detectable effect on improving clinical symptoms and urine quality. The clinically actionable symptoms included: elevated body temperature; increased frequency or discomfort due to bladder spasms; increased lower body tone, rigor, or spasticity; aggravated pain in the abdomen or change in its quality; dizziness; and headache. The symptoms relevant to urine quality were: change in its smell or appearance, or increase in sediment/white discharge [70].

Further studies focusing on the efficacy of intravesical or intravaginal probiotic use in recurrent UTIs prevention are needed. Presumably, these interventions could be considered in a specific group of pediatric patients, especially those at high risk of recurrent UTIs, and might be a part of a more individualized therapeutic approach.

#### 5.2.3. Intravesical Inoculation with Less-Pathogenic Bacteria

Alternative methods of UTIs prevention are investigated for patients at high risk of morbidity resulting from urinary tract infections, particularly those with neuropathic or neurogenic bladder.

The Cochrane Intervention Review by Toh et al. focused on the beneficial and harmful potential of probiotics in symptomatic UTI prevention in adults and children with neuropathic bladder. The review includes a total of three studies that assessed the intravesical instillation of a low virulent *E. coli* strain in lowering the risk of symptomatic UTI in patients with neuropathic bladder, predominantly from spinal cord injury. The authors concluded that the benefit of bladder inoculation with low-virulent strains of *E. coli* is uncertain because of the low confidence of the evidence (3 studies, 110 participants: RR=0.32, 95% CI: 0.08–1.19) [71].

### 5.3. Intravesical Instillations of Glycosaminoglycans

The possible benefit of intravesical administration of glycosaminoglycans in the prophylaxis of urinary tract infection is based upon the observation that loss of the glycosaminoglycan layer of the urothelium may increase bacterial adherence [51]. The glycosaminoglycan layer of the bladder wall is composed of negatively charged polysaccharides such as hyaluronic acid, heparin sulfate, dermatan sulfate, chondroitin sulfate, and keratan sulfate that tightly bound water and form a biofilm and provide an anatomical and functional barrier [51]. The literature evidence on the effectiveness of intravesical instillations of glycosaminoglycans in pediatric patients with recurrent UTIs is limited to two studies. The first study evaluating the impact of intravesical hyaluronic acid instillation on reduction of recurrent UTIs in a group of fifteen children was a case-series study by Fidan et al. The intervention consisted of four weekly sessions of intravesical hyaluronic acid instillation. The authors observed an overall complete or partial response to intravesical hyaluronic acid administration in 86.7% of children with complicated and uncomplicated recurrent UTIs at the end of the 24-month follow-up [72].

Cicek et al. aimed to assess the efficacy and safety of hyaluronic acid administered intravesically in reducing the rate of recurrent UTIs in ten pediatric patients with spina bifida and neurogenic bladder who perform clean intermittent catheterization. Hyaluronic acid was instilled intravesically weekly for four weeks, then monthly for three months. During the follow-up of 16 months, a significant decrease in mean UTIs per patient-month after the treatment in the study group was observed [73].

### 5.4. Immunostimulants, Vaccines, Autologous Bacterial Lysates

Immunostimulants and vaccines are supposed to exert their effects by activating the innate and adaptive immune systems [51]. Immunization with a mix of inactivated or lysed microorganisms is used to induce a protective immune response against uropathogenes. Examples of preparations for recurrent UTIs prevention with short information are presented in Table 2.

There are several technical and clinical difficulties associated with developing a vaccine against UTI: the mechanisms that induce protective immunity in the urinary tract are not fully understood; there is a large heterogeneity of uropathogenic *E. coli* strains; the patient subpopulations that would benefit from a vaccine are diverse [75]. Because uropathogenic *E. coli* strains utilize different virulence factors, an effective vaccine should provide a protective immune response against virulence factors that are expressed during colonization, invasion, and the formation of bacterial reservoirs [74].

Aziminia et al. conducted a systematic review regarding the effectiveness of vaccines or immunostimulants in recurrent UTIs prevention in adults. They included 10 randomized placebo-controlled trials and studied three vaccines: Uro-Vaxom, Urovac, and ExPEC4V. The use of vaccines showed to reduce UTI recurrence compared to placebo (RR = 0.74, 95% CI: 0.67–0.81) [76].

Another promising alternative in the treatment of recurrent UTIs is autologous immunotherapy. Autovaccines are bacterial lysates manufactured with the isolated microorganism from the culture of the infected site, inactivated by heat, and homogenized in a suspension, favoring IgG and IgM production and activation of lymphocyte T [79,80]. The study by Ahumada-Cota et al. showed that the autologous bacterial lysates improved the treatment’s effect and decreased the rate of recurrent UTIs in adults [79].

Hernández-Chiñas et al. conducted a prospective study in a group of 27 children with recurrent UTI due to congenital anomalies of the urinary tract after surgical correction with insufficient response to antimicrobial therapy. For patients who presented ≥10^5^ UFC/mL, an autologous bacterial lysate was manufactured using the isolated strain from the patient urine culture and was administered orally for a month. The use of autologous immunotherapy demonstrated a significant reduction in the presence of *E. coli* in the urine culture from 92.5% of patients at the beginning of the study, to 55.5% and 34% for the second and third months, respectively. This intervention controlled the infection for almost one year in more than 60% of observed children [80].

## 6. Conclusions

The microbiome of the urinary tract plays a significant role in maintaining health. The colonization of the urinary tract and stabilization of the urobiome takes place in childhood and alters with the child’s age. Dysbiosis of the urobiome may result in urological diseases, including recurrent urinary tract infections. The growing antibiotic resistance of bacteria encourages seeking non-antibiotic treatment options, which include manipulation of the microbiome. Reshaping the urobiome may help in control for recurrent UTIs and be an important alternative for long-term antibiotic therapy.

Different patient subgroups might benefit from different strategies of microbiome manipulation. Therefore, the choice of the optimal option should be individualized and adequate to the clinical situation of the child with recurrent UTIs. Cranberry products and oral probiotics might be beneficial in almost all children with recurrent UTIs. However, intravesical instillation of probiotics, hyaluronic acid, and immunostimulants should be considered as alternative or adjuvant therapy in patients with complicated recurrent UTIs, with neurogenic bladders, and at high risk of infection with multidrug-resistant bacteria. The possible effect of microbiome manipulation on the reduction of recurrent UTIs in children seems promising. An individualized combination of these strategies might result in the optimal clinical outcome. However, due to the uncertain effect of some interventions in the pediatric population, methodological challenges, relatively small sample size, further studies of urobiome in the prevention of urological diseases in children are needed. For clinical settings, future studies should aim to establish the relationship between the urobiome and the most common UTI risk factors in children, such as vesicoureteral reflux, obstructive uropathy, overactive bladder, or neurogenic bladder. Ideally, the studies should be longitudinal, and the study group should be representative. Deeper insight into host-microbial interactions and their association with different urinary tract pathologies, defining models for UTI in patients with particular comorbidities might enable the development of individualized preventive methods dedicated to specific groups of patients. Bearing in mind that the urobiome is not only bacteria, future research should also encompass the urinary tract virome and investigate the role of bacteriophages.

## Figures and Tables

**Table 1:** UTIs classification according to complicating factors [40,41].

UTI	Characteristic	Related Microorganisms
uncomplicated	occurs in immunocompetent patients with anatomically and functionally normal urinary tract	uropathogenic *E. coli* (causative factor in 80%)
complicated	occurs in patients with anatomical or neurological abnormalities of urinary tract (i.e., hydronephrosis, vesicoureteral reflux), in patients with compromised immunity, or if foreign bodies are present in patient’s urinary tract (i.e., calculi, catheters)	*E. coli* (often presenting combination of many virulence factors, or multi-drug resistant profile), non-*E. coli* infection (i.e., *Klebsiella* spp., *Enterococcus* spp., *Pseudomonas aeruginosa*)

**Table 2:** Bacterial immunostimulants against urinary tract infection [51,74,75,76,77,78].

Preparation	Composition	Administration Route	Manufacturer
Strovac (Solco-Urovac) Urovac	six uropathogenic *E. coli* strains, 1 strain of each *Proteus mirabilis*, *Klebsiella pneumoniae*, *Morganella morganii* and *Enterococcus faecalis*	intramuscular (Strovac), vaginal vaccine (Urovac)	Strathmann GmbH (Strovac) Solco Basel Ltd. (Urovac)
Uro-Vaxom (OM89)	membrane proteins of 18 strains of uropathogenic *E. coli*	oral capsule	OM Pharma
Uromune (MV140)	*E. coli*, *Klebsiella pneumoniae*, *Proteus vulgaris*, *Enterococcus faecalis*	sublingual	Inmunotek (clinical research)
Urostim/Urvakol	*E. coli*, *Klebsiella pneumoniae*, *Proteus vulgaris*, *Enterococcus faecalis*	oral tablets	BB-NCIPD Ltd. (clinical research)
ExPEC4V	four *E. coli* O-antigens (O1A, O2, O6A, O25B) conjugated to exotoxin protein A	intramuscular	GlycoVaxyn AG (clinical research)
CP923	developed using the mutations in capsule and O antigen from LPS in uropathogenic *E. coli* strain	intranasal	pre-clinical research
